# Intracoronary Gene Delivery of the Cytoprotective Factor Vascular Endothelial Growth Factor-B_167_ in Canine Patients with Dilated Cardiomyopathy: A Short-Term Feasibility Study

**DOI:** 10.3390/vetsci6010023

**Published:** 2019-03-06

**Authors:** Paola Paradies, Lucia Carlucci, Felix Woitek, Francesco Staffieri, Luca Lacitignola, Luigi Ceci, Daniela Romano, Mariateresa Sasanelli, Lorena Zentilin, Mauro Giacca, Stefano Salvadori, Antonio Crovace, Fabio A. Recchia

**Affiliations:** 1 Department of Emergency and Organ Transplantation, Section of Veterinary Clinics and Animal Production; University of Bari, 70010 Bari; Italy; francesco.staffieri@uniba.it (F.S.); luca.lacitignola@uniba.it (L.L.); luigi.ceci@uniba.it (L.C.); dadda.romano@gmail.com (D.R.); mariateresa.sasanelli@uniba.it (M.S.); antonio.crovace@uniba.it (A.C.); 2 Institute of Life Sciences, Scuola Superiore Sant’Anna, 56100 Pisa, Italy; lucia.carlucci3@gmail.com (L.C.); f.recchia@santannapisa.it (F.A.R.); 3 Heart Center, Dresden at the Technical University of Dresden, 01067 Dresden, Germany; felix.woitek@mailbox.tu-dresden.de; 4 Molecular Medicine Laboratory, International Centre for Genetic Engineering and Biotechnology (ICGEB), 34149 Trieste, Italy; lorena@icgeb.org (L.Z.); giacca@icgeb.org (M.G.); 5 CNR, Institute of Clinical Physiology, Area della Ricerca, 56121 Pisa, Italy; stefsa@ifc.cnr.it

**Keywords:** clinical study, intracoronary cytoprotective gene therapy, dogs

## Abstract

Dilated cardiomyopathy (DCM) is a myocardial disease of dogs and humans characterized by progressive ventricular dilation and depressed contractility and it is a frequent cause of heart failure. Conventional pharmacological therapy cannot reverse the progression of the disease and, in humans, cardiac transplantation remains the only option during the final stages of heart failure. Cytoprotective gene therapy with vascular endothelial growth factor-B_167_ (VEGF-B_167_) has proved an effective alternative therapy, halting the progression of the disease in experimental studies on dogs. The aim of this work was to test the tolerability and feasibility of intracoronary administration, under fluoroscopic guidance, of VEGF-B_167_ carried by adeno-associated viral vectors in canine DCM patients. Ten patients underwent the gene delivery procedure. The intraoperative phase was well tolerated by all dogs. Clinical and echocardiographic assessments at 7- and 30-days post-procedure showed stable conditions compared to the pre-procedure phase. The results of this work indicate that intracoronary VEGF-B_167_ gene delivery is feasible and tolerated in dogs with DCM. Further monitoring/investigations are ongoing to evaluate the effects of this therapy on disease progression.

## 1. Introduction

Dilated cardiomyopathy (DCM) is a myocardial disease of dogs and humans characterized by progressive ventricular dilatation and systolic dysfunction. It is usually associated to arrhythmias and risk of sudden death and is a frequent cause of heart failure. Epidemiological studies in humans report that 5.3 million people over 20 years of age are affected by DCM in the USA alone [1]. DCM constitutes a serious clinical problem also in dogs, affecting mostly giant and large breeds [2,3].

Current pharmacological therapies can mitigate symptoms of heart failure and arrhythmias, while they cannot reverse the progression of the disease. In dogs, pimobendan, an inotropic agent (not registered for humans) is proving efficacious in increasing the survival time [4,5,6,7]. Nevertheless, the available pharmacological agents are unable to stop myocardial degeneration, thus the disease progression, both in humans and in dogs. In humans, cardiac transplantation remains the only option during the final stages of heart failure, with all the involved risks and costs.

In recent years the interest of researchers has been focused on new strategies based on modern biotechnologies, such as gene and cellular therapy [8,9], to directly cure the myocardium rather than the consequences of heart failure. The dog represents an excellent model to study human DCM, that is why numerous pre-clinical and clinical studies on the efficacy of innovative therapies to treat DCM are performed in dogs [9,10,11,12,13,14].

Among potential cardioprotective factors, we have investigated the vascular endothelial growth factor-B (VEGF-B), due to its marked cytoprotective and anti-apoptotic action and no pro-angiogenic effects [15]. These properties render it particularly well-suited for gene therapy of non-ischemic dilated cardiomyopathy, in which the increased rate of apoptosis seems to play a major role [16,17,18]. The isoform VEGF-B_167_, delivered as a transgene encapsidated into adeno-associated viruses 9 (AAV-9), has proved an effective therapy to treat DCM, halting the progression of the disease in experimental pre-clinical studies in rodents and dogs [8,19,20]. AAV vectors have an excellent safety profile, are poorly immunogenic and sustain long-term expression of the carried transgene [21].

The efficacy of the cytoprotective gene therapy in dogs with experimental tachypacing-induced dilated cardiomyopathy has been initially tested by direct intramyocardial injection [20] and later by intracoronary infusion [8], which is more acceptable in the clinical practice. However, no data are available to date on the feasibility and efficacy of cytoprotective gene therapies in canine patients presenting with naturally acquired DCM and signs of fully developed heart failure.

Here we report the first phase of an ongoing larger clinical trial aimed at testing, in dogs with naturally acquired cardiomyopathy and heart failure, the feasibility and tolerability of intracoronary gene therapy with VEGF-B_167_.

## 2. Materials and Methods

### 2.1. Patients Selection and General Protocol

Dogs with diagnosis of familiar DCM based on breed predisposition, clinical history and radiographic, echocardiographic and electrocardiographic evaluation [3,22], with or without atrial fibrillation and dogs with hypokinetic dilatative cardiomyopathy (DCM-like) associated to atrial fibrillation were considered eligible for inclusion. Given the aim of the study and the important open questions about the safety of intracoronary gene delivery in patients with heart failure, we chose to include only symptomatic dogs (dogs with at least one sign of heart failure referred in the clinical history). Eligible dogs underwent clinical examination and blood sampling for haemato-biochemical analysis, thyroid hormone profile, antigenic test for *Dirofilaria immitis* and troponin. The presence of concomitant diseases such as hypothyroidism, dirofilariasis, acute myocarditis, pulmonary hypertension, diabetes, myasthenia gravis and pregnancy were exclusion criteria. Dogs under standard medical treatment (furosemide, spironolactone, Pimobendan, ACE-I and anti-arrhythmics) were not excluded, the medical protocol was revisited to obtain the best clinical control and uniformed for all the patients at least one month before gene delivery.

At D0 (day of coronary catheterization) the enrolled dogs underwent trans-thoracic echocardiography, ECG (using an ESAOTE P8000, ESAOTE, Genoa, Italy) recording and blood sampling for laboratory routine tests. The dog owners gave informed written consent for the intracoronary therapy, after explanation of the procedure and of its possible risks. The procedures for this study were approved by the Italian Ministry of Health (Ministry Authorization Number 180946122, May 2016).

At the end of the catheterization procedure, dogs were clinically and instrumentally monitored for at least 12 h after recovery from general anaesthesia. All adverse events occurring during hospitalization were recorded. The owners were instructed to report any other adverse event appearing during the study period.

Clinical and instrumental evaluations were performed at day 7 (D7) and 30 (D30) after the procedure, using the same equipment. All pre- and post-procedure echocardiographic values, haematobiochemical exams and ECG abnormalities were compared.

### 2.2. Echocardiographic Exams

A trans-thoracic echocardiography was performed in right and left lateral recumbency on non-sedated patients at D0 (pre-procedure), D7 and D30 using an Esaote MyLab-alpha system equipped with a 2–4 MHz cardiac probe. The following parameters of left ventricular function were calculated according to standard methods: ejection fraction (EF) and end-systolic volume index (ESVI) by Simpson in B mode from left apical four chamber view and EF Teichholz and fractional shortening (FS) in M mode at papillary muscle level. Cardiac remodelling and left ventricular volume overload were assessed by measuring and/or calculating: sphericity index (SI), end point to septal separation (EPSS), mitral and tricuspidal regurgitant jets and left ventricular end-diastolic volume index (EDVI). Furthermore, the left atrium to aorta diastolic diameter ratio (LA/Ao) and the diastolic pattern from mitral flow in PW were evaluated. To reduce the expected interference of atrial fibrillation, measurements were repeated three times over 5 consecutives cycles.

Furthermore, parameters of radial, circumferential and longitudinal (global) strain were acquired through video clips (obtained concurrently with the acquisition of the scans necessary for the standard echocardiographic examination), as well as the strain parameters of EF and fraction of shortening (FAC). All analyses (M-mode, B-mode, Doppler and Speckle Tracking) were performed off-line.

### 2.3. Viral Vector Production

The transgene VEGF-B was encapsidated into adeno-associated virus of serotype 9 (AAV9) at the Molecular Medicine Laboratory of the International Centre for Genetic Engineering and Biotechnology (ICGEB) in Trieste, Italy, sent to the hospital in dry ice and maintained at −80 °C till the procedure. AAV9 was chosen because of its known cardiotropism [21]. In brief, mouse VEGF-B167 cDNA was incorporated into recombinant AAV-9 prepared by the AAV Vector Unit at ICGEB Trieste. We have previously used mouse VEGF-B transgene in experimental dog models [8,20]. We found it very efficacious and it also offers the advantage of being distinguishable from endogenous canine VEGF-B when searched by PCR in explanted tissues, post-mortem. Methods for AAV production and purification were previously described [23]. AAV titres were in the range of 1 × 1014 genome copies per millilitre.

### 2.4. Anesthetic Protocol and Intra-Operative Monitoring

Dogs were sedated with 0.3 mg/kg methadone intramuscularly; after premedication with lidocaine (1 mg/kg, intravenously), general anaesthesia was induced by midazolam (0.3 mg/kg) and Propofol 3 mg/kg intravenously. After intubation, anaesthesia was maintained with 1–1.2% isoflurane diluted in oxygen, while lidocaine was continuously infused (30–50 mcg/kg/min) to prevent major arrhythmias. Positive pressure ventilation was maintained during the procedure.

The following cardiovascular and respiratory parameters (Datex Ohmeda S/5^®^, Datex Ohmeda, Helsinki, Finland and Mostcare^®^, Vygon, Padova, Italy) were monitored: heart rate and ECG, arterial pressure (via intra-arterial catheter), stroke volume and cardiac output, respiratory rate, end-tidal CO_2_, haemoglobin oxygen saturation, tidal volume and airway pressure.

0.9% saline solution was continuously infused at the rate of 3 mL/kg/h. The goal was to maintain mean arterial pressure (MAP) between 60 and 70 mmHg. In case of MAP values lower than 60 mmHg, fluid infusion was increased to 5 mL/kg/h. In case of MAP values lower than 50 mmHg and in patients unresponsive to saline infusion, colloids (gelatins, infuplas^®,^ Fresenius Kabi, Italy) were administered in boluses of 1 mL/kg, up to a maximum of 5 mL/kg. In addition, dobutamine infusion was started in a range between 0.5 and 2 mcg/kg/min, when required, if dogs were not responsive to any fluid infusion. During catheterization of the coronary artery, 0.3 mg/kg of rocuronium, a short-acting neuromuscular blocker, was administered in bolus to obtain the complete immobility of the patient.

At the end of the procedure, patients were kept under general anaesthesia and fully monitored for at least one hour to intervene in case of any major adverse event, which was immediately treated based on current guidelines.

The recovery from general anaesthesia was supported by fluid infusion and warming devices. All dogs were kept under 5 cm H_2_O of continuous positive airway pressure (CPAP) during recovery by means of a helmet CPAP system, until adequate oxygenation and respiratory pattern were reached.

All dogs received 22 mg/kg of cefazoline every 1.5 h intraoperatively and thereafter every 8 h for 24 h. In case signs of pain or discomfort were detected, 0.010 mg/kg IM of buprenorphine was administered intramuscularly.

### 2.5. Intracoronary Gene Delivery Procedure

After positioning the patient in right lateral recumbency, a 6 F sheath was inserted percutaneously into the right femoral artery for coronary catheterization. Left circumflex and anterior descending coronary arteries were selectively catheterized under fluoroscopic guidance. Iodixanol in electrolytic formulation (Visipaque®, GE Healthcare, Milan, Italy) was used as contrast medium.

When a 2.5 F micro-infusion catheter was positioned in coronary, a 20 mL suspension containing AAV-VEGF-B_167_ plus 3 ng/kg of adenosine, 5 ng/kg of substance P and 1 mg/kg/min of nitro-glycerine in 0.9% saline solution was rapidly prepared. Adenosine, substance P and nitro-glycerine were used to increase the permeability in myocardial capillaries. The suspension was administered with an infusion pump over 20 min, followed by 10-min flushing with saline solution. At the end of this procedure, the catheter was removed and the femoral artery was closed using a vascular closure device (6F AngioSeal^TM^, Terumo, Rome, Italy).

### 2.6. Post-Procedural Monitoring

Post-procedural monitoring was defined as the time from recovery from general anaesthesia to discharge, which occurred when dogs were deemed clinically and electrocardiographically stable. After discharge, the same pharmacological therapy they were receiving before procedure was resumed for all dogs and continued at home. In addition, 20 mg/kg ceftriaxone was administered twice a day for 6 days after surgery. Dogs were monitored for ECG and arterial blood pressure and cardiac output for at least 12 h after the procedure. Blood electrolytes (Na, K, Cl) were also monitored.

### 2.7. Statistical Analysis

Descriptive analyses were expressed as mean and standard deviation or percentages as appropriate.

Echocardiographic and laboratory data was analysed by analysis of variance (ANOVA) for repeated measures to compare means at D0, D7 and D30. P-values less than 5% were considered statistically significant. Bonferroni correction was applied in pairwise comparisons.

Statistical analysis was performed using GraphPad PRISM version 8.0.0 (131) for Windows, GraphPad Software (www.graphpad.com).

## 3. Results

Ten patients underwent the gene delivery procedure. The selected group included 2 Great Danes, 1 German Shepard, 1 Giant Schnauzer, 1 Dobermann, 1 Saint Bernard and 4 molossoids. They were all males except for one female, with a mean age of 6.65 ± 1.76 years, body weight of 49.75 ± 14.35 kg and affected by familiar DCM or DCM-like cardiomyopathy. All dogs except for two were in atrial fibrillation. In one dog (Dobermann) ventricular premature complexes (VPCs) were recorded on a sinus rhythm background. Signalment, clinical history as well as clinical signs observed after pharmacological treatment and ECG features at T0 are reported in Table 1. In brief, only one dog (C4) with severe disease refractory to treatment underwent the procedure while in congestive heart failure. Echocardiographic exams at D0 showed signs of generalized or segmental hypokinesia associated to cardiac remodelling in all dogs.

The intraoperative phase was well tolerated by all patients. During intracoronary AAV-VEGF-B167 infusion, no adverse events were observed except for the expected hypotension [6] that was pharmacologically treated as reported in Materials and Methods. Sporadic VPCs, not present at baseline, appeared in all dogs during the catheterization phase but disappeared immediately after catheter retraction. All animals had an uneventful recovery from anaesthesia. In 3 dogs (30%) rhythm abnormalities developed at 3 to 6 h after recovery from anaesthesia (Table 2).

**Figure 1:**
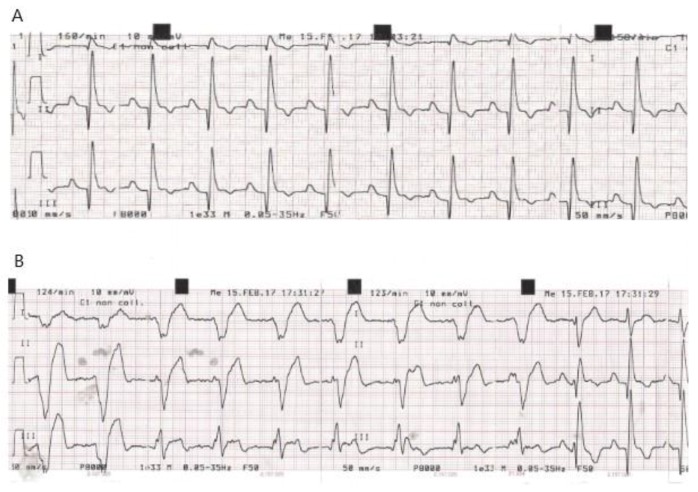
Dog C4 (Great Dane M, 6.5 years old). (**A**) ECG recorded at D0 before gene delivery procedure showing a sinus rhythm. (**B**) ECG recorded at 5 h post procedure.

**Figure 2:**
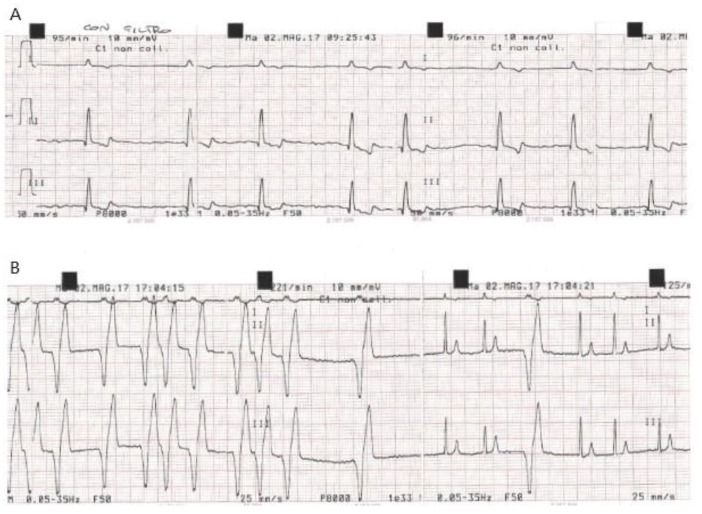
Dog C5 (Saint Bernard M, 6 years old). (**A**) ECG recorded at D0 before gene delivery procedure. (**B**) ECG recorded at 5 h post procedure.

**Figure 3:**
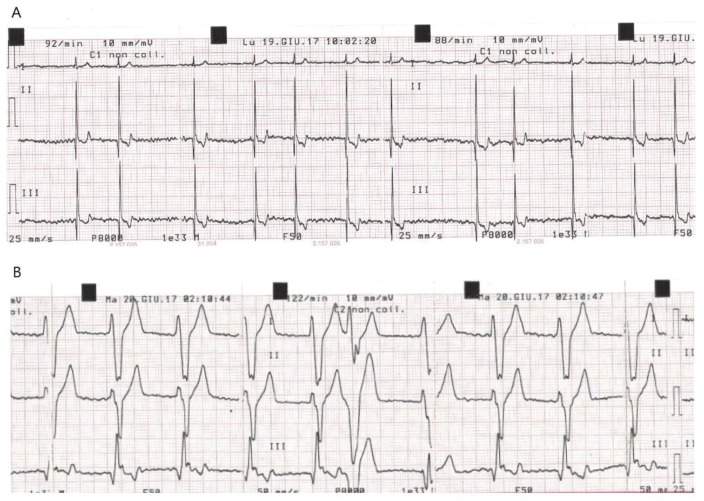
Dog C10 (Schnauzer M, 8 years old). (**A**) ECG recorded at D0 before gene delivery procedure. (**B**) ECG recorded at 5 h post procedure.

In particular, in these dogs (two in atrial fibrillation and one in sinus tachycardia at baseline) a variable ventricular rhythm developed and spontaneously resolved over at least 48 h with no need for treatment (Figure 1, Figure 2 and Figure 3).

One of these dogs (C4) also showed hypopotassaemia and needed intravenous potassium supply. Discharge was authorized 48 h after the procedure. The other dogs did not show any abnormalities during post procedural monitoring and were sent home after 12 h of observation. All dogs were able to walk and drink before discharge.

The owners of the treated dogs did not report any adverse events following discharge, except for one. This dog developed a hematoma at the internal side of the posterior right limb (site of catheter introduction) that resolved spontaneously one week later.

**Figure 4:**
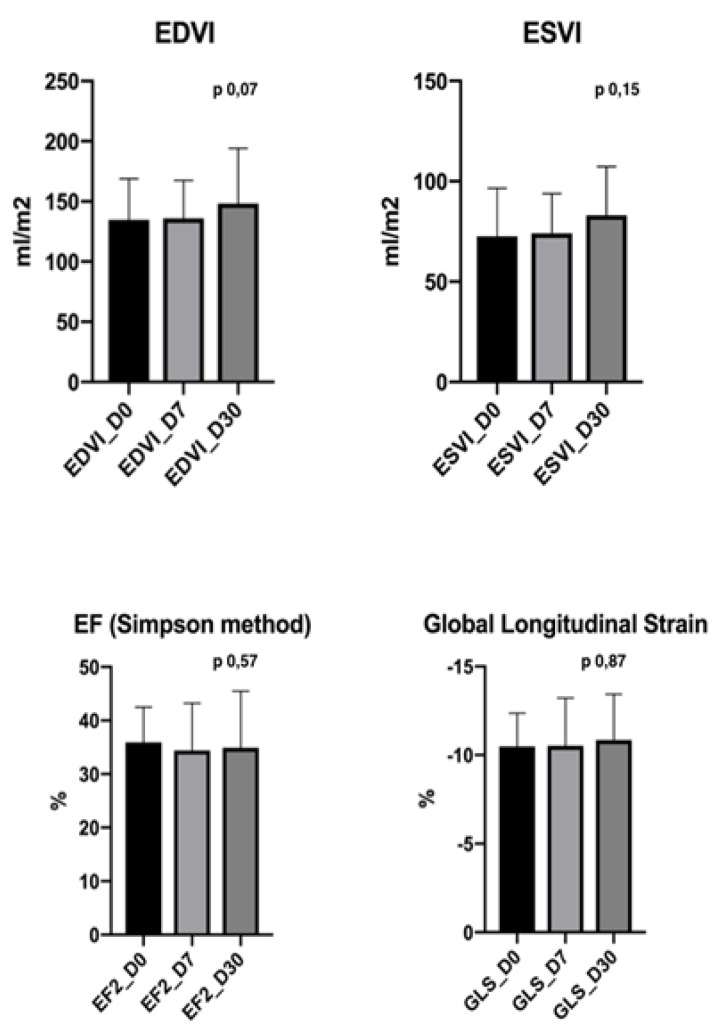
Both conventional and strain echocardiographic parameters remained stable throughout the study, without significative differences pre- and post-procedure, indicating the absence of direct adverse repercussions on heart function.

Clinical assessments at D7 showed, in all treated dogs, stable conditions similar to those observed before the procedure. The ECG at D7 was also not changed compared to the pre-procedure (D0) phase, indicating the absence of residual adverse events and the complete recovery after coronary catheterization. The laboratory exams as well as echocardiographic parameters (standard and strain) recorded at D7 and D30 did not show any statistical differences compared to D0 (Table 3 and Figure 4).

## 4. Discussion

The results of this study indicate that intracoronary delivery of transgenes carried by AAV-9 is feasible and tolerated in canine patients with familiar DCM or DCM-like disease. Both of the mini-invasive procedure of coronary artery catheterization and the infusion of AAV-VEGF-B_167_ suspended in a solution containing adenosine, substance P and nitro-glycerine were tolerated in dogs with naturally occurring disease, as previously reported in dogs with experimentally induced DCM [8]. All dogs displayed an uneventful recovery from anaesthesia; at one week after the procedure their clinical and haemato-chemical condition were similar to those found during the pre-procedure phase and remained stable also at D30. Furthermore, parameters from conventional and strain echocardiography do not show significant differences throughout the study period to indicate the absence of direct adverse events on heart function after the gene delivery procedure.

Cardiac rhythm abnormalities detected during the post-procedural monitoring were resolved within 48 h, with no need for pharmacological treatment. They occurred hour after the procedure, hence they could not be referred to myocardial mechanical irritation caused by catheterization [24], differently from the sporadic VPCs that appeared during the intraoperative phase and spontaneously resolved soon after catheter withdrawal. Moreover, they could not be attributed to VEGF-B_167_, since the expression of transgenes carried by AAV typically takes approximately 10 days [19]. On the other hand, VEGF-B_167_ was likely expressed at D7 and certainly expressed at D30, nonetheless no major alterations were observed.

In literature, rhythm abnormalities associated to transcatheter procedures have been related to the type and the duration of the exposure to the contrast medium and fluoroscopic procedure, the pro-arrhythmic effect of specific devices and the inflammatory response involving the conduction system [24,25,26]. Considering that the contrast medium used in this study (Visipaque®) was demonstrated to be minimally arrhythmogenic [27] and that the duration of the procedure was relatively short in all dogs (less than 2 h), we speculate that rhythm abnormalities observed during post-procedure monitoring were the consequence of a transient and small inflammatory reaction of the myocardium to the inoculation of the viral suspension, perhaps involving the conduction system.

Of note, our data support the feasibility and tolerability of this intervention also in dogs at an advanced stage of heart failure, with or without atrial fibrillation, since our population included symptomatic dogs with signs of cardiac remodelling.

We previously showed in experimental dogs with compensated heart failure and initial cardiac remodelling that intracoronary administration of AAV-VEGF-B_167_ was able to delay the progression towards decompensation [8]. Unfortunately, the model we used is an accelerated form of DCM, not suited to test long-term effects of therapy, especially if the treatment is initiated when heart failure has already developed. For this reason, the long term follows up of this study will provide precious, albeit still partial information about the real efficacy of VEGF-B_167_ in dogs with the naturally occurring DCM.

At least two important limitations of this study should be acknowledged. Firstly, the sample size is small, due to the formidable difficulties associated with the recruitment of this type of patients for such an invasive procedure, very uncommon in the veterinary practice. Secondly, this study could not be blinded, especially because there is no control arm with infusion of AAVs carrying ineffective DNA. Notwithstanding these limitations, perhaps unavoidable if we consider that very few studies have tested cardiac gene therapy in dogs with spontaneous DCM [28], the initial evidence of safety is per se very encouraging. Long term results, if positive, will pave the way for larger clinical trials in dogs and humans as well.

## Figures and Tables

**Table 1:** Signalment and clinical history of enrolled dogs. Clinical signs, cardiac rhythm, heart rate and standard treatment ongoing at time of procedure (T0) are reported for each dog included in the study.

Dog	Breed	Age (Years)	Sex	Clinical History	Clinical Signs and Symptoms at T0	ECG Findings and Heart Rate at T0	Ongoing Treatment (from at Least One Month before Procedure)
C 1	Italian Mastiff	3	M	Disorexia, exercise intolerance, ascites, Afib	None. Afib DCM-like	Afib, HR = 134/min.	pimobendan 0.25 mg/kg q12 h, digossina 0.22 mg/m^2^ q12 h, benazepril 0.25 mg/kg q12 h, furosemide 2 mg/kg q12 h
C 2	Dogue de Bordeau	8	M	Mild depression, ascites, Afib	None. Afib DCM-like	Afib, HR = 121/min.	pimobendan 0.25 mg/kg q12 h, digossina 0.22 mg/m^2^ q12 h, benazepril 0.25 mg/kg q12 h
C 3	German Shepherd	8	M	Ascites, Afib	None. Afib DCM-like	Afib, HR = 147/min.	pimobendan 0.25 mg/kg q12 h, digossina 0.22 mg/m^2^ q12 h, benazepril 0.25 mg/kg q12 h, furosemide 2 mg/kg q24 h
C 4	Great Dane	6.5	M	Depression, exercise intollerance, ascites	Exercise intollerance, ascites, weak pulse. Familiar DCM	Synus rhythm HR = 160/min.	pimobendan 0.25 mg/kg q12 h, torasemide 0.3 mg/kg q12 h, benazepril 0.25 mg/kg q12 h, spironolattone 2 mg/kg q24h
C 5	Saint Bernard	6	M	Syncope, both thoracic and abdominal effusion, Afib	None. Afib Familiar DCM	Afib, HR = 95/min.	pimobendan 0.25 mg/kg q12 h, digossina 0.22 mg/m^2^ q12 h, benazepril 0.25 mg/kg q12 h, furosemide 2 mg/kg q12 h
C 6	Dobermann Pincher	5	F	Syncope	None. Familiar DCM	Synus rhythm, WP, HR = 51/min, sporadic PVC (1/60”)	pimobendan 0.25 mg/kg q12 h, benazepril 0.25 mg/kg q12 h, furosemide 2 mg/kg q12 h sotalol 2 mg/ q12 h
C 7	Italian Mastiff	7.5	M	Exercise intollerance, ascites, Afib	None. Afib DCM-like	Afib, HR = 88/min.	digossina 0.22 mg/m^2^ q12 h, benazepril 0.25 mg/Kg q12 h, furosemide 2 mg/kg q12 h
C 8	Amstaff	7	M	Acute depression, ascites, Afib	None. Afib DCM-like	Afib, HR = 150/min	pimobendan 0.25 mg/kg q12 h, benazepril 0.25 mg/Kg q12 h, digossina 0.22 mg/m^2^ q12 h, furosemide 2 mg/kg q12 h
C 9	Great Dane	5.5	M	Syncope, dyspnoea post-surgery, Afib	None. Afib Familiar DCM	Afib, HR = 150/min	pimobendan 0.25 mg/kg q12 h, benazepril 0.25 mg/Kg q12 h, digossina 0.22 mg/m^2^ q12 h, furosemide 2 mg/kg q12 h
C 10	Giant Schnauzer	8	M	Exercise intollerance, Afib	None. Afib Familiar DCM	Afib, HR = 85/min	pimobendan 0.25 mg/kg q12 h, digossina 0.22 mg/m^2^ q12 h, benazepril 0.25 mg/Kg q12 h, furosemide 2 mg/kg q12 h

**Table 2:** New conduction abnormalities appearing in the post-procedure monitoring in the 10 dogs after intracoronary cytoprotective gene therapy. ECG abnormalities at baseline, presence of new abnormalities (compared with baseline) at post-procedure monitoring and at D7 are reported. Furthermore, the time of discharge and the potassium serum concentration at the time of weak up after anaesthesia is reported.

Dog	ECG at Baseline (T0)	New Conduction Abnormalities Appeared in the PPM (First Few Hours during Post-Anaesthesia Recovery)	Time of Discharge	ECG at D7 Compared to D0:	K^+^ Serum Concentration (mEq/L) at Weak Up
C 1	Afib, HR = 134/min	No changes	12 h	No changes	n.d.
C 2	Afib, HR = 121/min	No changes	12 h	No changes	n.d.
C 3	Afib, HR = 147/min	No changes	12 h	No changes	K^+^ = 4.1
C 4	Synus rhythm HR = 160/min	VR (5h)	48 h	No changes	K^+^ = 3
C 5	Afib, HR = 95/min	Sporadic VPC (4h) exiting in VR (6h)	48 h Sporadic monomorphic VPCs	Residual sporadic monomorphic VPCs	K^+^ = 4.4
C 6	Synus rhythm, WP, HR = 51/min, sporadic PVC	No changes	12 h	No changes	K^+^ = 4
C 7	Afib, HR = 88/min.	No changes	12 h	No changes	K^+^ =3.6
C 8	Afib, HR = 150/min	No changes	12 h	No changes	K^+^ = 4.2
C 9	Afib, HR = 150/min	No changes	12 h	No changes	K^+^ = 4.3
C 10	Afib, HR = 85/min	Afib + VPC (2h) exiting in VR (4h).	48 h	No changes	K^+^ = 4.2

Legend: PPM = post procedure monitoring time; Afib = atrial fibrillation; HR= heart rate; VPCs = ventricular premature complexes.

**Table 3:** Echocardiographic parameters (standard and strain) recorded at D0 and D30.

Dog	EF (%) D0	EF (%) D30	EDVI (mL/m^2^) D0	EDVI (mL/m^2^) D30	ESVI (mL/m^2^) D0	ESVI (mL/m^2^) D30	GLS (%) D0	GLS (%) D30
C 1	21	19	79	93	51	61	7,7	12,1
C 2	38	40	98	100	53	52	9,6	9,7
C 3	36	35	186	213	82	122	10,7	9,9
C 4	19	22	163	181	115	130	9,4	9,8
C 5	46	46	133	150	76	78	9,7	10,1
C 6	28	29	155	153	105	110	10,6	12,1
C 7	38	36	106	105	52	64	11,4	11,6
C 8	44	44	189	195	88	100	11,6	10,9
C 9	36	39	193	182	90	110	10,2	12,2
C 10	38	39	137	137	79	69	10,1	8,27

Legend: EF = ejection fraction measured with the Simpson’s method; EDVI end-diastolic volume index; ESVI end-systolic volume index; GLS global longitudinal strain.
